# Consensus communication on early peanut introduction and the prevention of peanut allergy in high-risk infants

**DOI:** 10.1186/s40413-015-0076-x

**Published:** 2015-08-03

**Authors:** David M. Fleischer, Scott Sicherer, Matthew Greenhawt, Dianne Campbell, Edmond S. Chan, Antonella Muraro, Susanne Halken, Yitzhak Katz, Motohiro Ebisawa, Lawrence Eichenfield, Hugh Sampson, FOR THE LEAP TRIAL TEAM, SECONDARY CONTRIBUTORS

**Affiliations:** American Academy of Allergy, Asthma & Immunology (AAAAI), Milwaukee, WI United States of America; American Academy of Pediatrics (AAP), Chicago, IL United States of America; American College of Allergy, Asthma & Immunology (ACAAI), Chicago, IL United States of America; Australasian Society of Clinical Immunology and Allergy (ASCIA), Brookvale, NSW Australia; Canadian Society of Allergy and Clinical Immunology (CSACI), Orleans, ON Canada; European Academy of Allergy and Clinical Immunology (EAACI), Zurich, Switzerland; Israel Association of Allergy and Clinical Immunology (IAACI), Tel-Hashomer, Israel; Japanese Society for Allergology (JSA), Tokyo, Japan; Society for Pediatric Dermatology (SPD), Indianapolis, IN United States of America; World Allergy Organization (WAO), Milwaukee, WI United States of America; Department of Pediatrics, Box 1198, Division of Allergy-Immunology, Jaffe Food Allergy Institute at the Icahn School of Medicine at Mount Sinai, One Gustave L. Levy Place, New York, NY 10029 USA

**Keywords:** Allergy prevention, Complementary feeding, Peanut allergy

## Abstract

The purpose of this brief communication is to highlight emerging evidence to existing guidelines regarding potential benefits of supporting early, rather than delayed, peanut introduction during the period of complementary food introduction in infants. This document should be considered as interim guidance based on consensus among the following organizations: American Academy of Allergy, Asthma & Immunology; American Academy of Pediatrics; American College of Allergy, Asthma & Immunology; Australasian Society of Clinical Immunology and Allergy; Canadian Society of Allergy and Clinical Immunology; European Academy of Allergy and Clinical Immunology; Israel Association of Allergy and Clinical Immunology; Japanese Society for Allergology; Society for Pediatric Dermatology; and World Allergy Organization. More formal guidelines regarding early-life, complementary feeding practices and the risk of allergy development will follow in the next year from the National Institute of Allergy and Infectious Diseases – sponsored Working Group and the European Academy of Allergy and Clinical Immunology.

## Introduction and rationale

Peanut allergy is an increasingly troubling global health problem affecting between 1 and 3 % of children in many westernized countries. Although multiple methods of measurement have been used and specific estimates differ, there appears to be a sudden increase in the number of cases in the past 10- to 15-year period, suggesting that the prevalence might have tripled in some countries, such as the United States. Extrapolating the currently estimated prevalence, this translates to nearly 100,000 new cases annually (in the United States and United Kingdom), affecting some 1 in 50 primary school-aged children in the United States, Canada, the United Kingdom, and Australia. A similar rise in incidence is now being noted in developing countries, such as Ghana [1–6].

The **purpose of this brief communication** is to highlight emerging evidence for existing allergy prevention guidelines regarding potential benefits of supporting early rather than delayed peanut introduction during the period of complementary food introduction in infants. A recent study, entitled “Randomized trial of peanut consumption in infants at risk for peanut allergy” demonstrated a successful 11–25 % absolute reduction in the risk of peanut allergy in high-risk infants (and a relative risk reduction of up to 80 %) if peanut was introduced between 4 and 11 months of age [7]. In light of the significance of these findings, this document serves to better inform the decision-making process for healthcare providers regarding such potential benefits of early peanut introduction. More formal guidelines regarding early-life, complementary feeding practices and the risk of allergy development will follow in the next year from the National Institute of Allergy and Infectious Diseases (NIAID)-sponsored Working Group and the European Academy of Allergy and Clinical Immunology (EAACI), and thus this document should be considered as interim guidance.

## Summary of new evidence

In the Learning Early About Peanut Allergy (LEAP) trial, 640 high-risk United Kingdom infants (See Box 1) between the ages of 4–11 months were randomized to consume peanut products at least three times a week (6 g of peanut protein; equivalent to 24 g peanuts or 3 teaspoons of peanut butter per week) or to completely avoid peanut products for the first 5 years of life. This included 542 infants found to have negative skin prick test (SPT) responses to peanut at study entry, and 98 infants with SPT wheal diameters to peanut of between 1 and 4 mm (minimally positive SPT response) at study entry. An additional 76 children were excluded from study entry before randomization based on an SPT response of greater than 5 mm, which was assumed to result in a very high likelihood of reacting to a peanut challenge. In an intention-to-treat analysis, 17.2 % in the peanut avoidance group compared to 3.2 % in the peanut consumption group had food challenge-proved peanut allergy by age 5 years, corresponding to a 14 % absolute risk reduction, a number needed to treat (NNT, eg, number of persons needed to be treated for one to receive benefit) of 7.1, and a relative risk reduction of 81 % [7].

When examined in further detail, the isolated beneficial effects for both the primary and secondary prevention of peanut allergy translated to an NNT of 8.5 among the infants with negative SPT responses and an NNT of 4 among the infants with minimally positive SPT responses. Secondary analyses also showed similar levels of prevention in white, black and Asian (Indian and Pakistani) children. Overall, the risk of early introduction in this group was low: 7 of the 319 children randomized to the consumption group reacted to peanut at the baseline food challenge, suggesting that peanut food challenges and introduction, even in infants with minimally positive SPT responses, are safe and feasible. Six children in the consumption group had peanut allergy during the study, indicating that peanut allergy can still develop despite attempts at primary and secondary prevention. Finally, the LEAP trial only included high-risk infants with a minimal or negative SPT response to peanut and therefore does not address a strategy for those without these risk factors for peanut allergy [7].

## How does the leap trial affect present guidance for early complementary feeding practices?

Existing guidelines pertaining to the early introduction of complementary foods have indicated that the introduction of highly allergenic foods, such as peanut, need not be delayed past 4 or 6 months of life. However, they do not actively recommend introduction of peanut between 4 and 6 months of age in high-risk infants, and some of these guidelines specify that certain infants considered at high risk for allergic disease are recommended to first consult an expert [8–14].

The LEAP data provide *Level 1* evidence that the practice of early peanut introduction is safe and effective in selected high-risk infants. This study is the first prospective, randomized trial of early peanut intervention and informs provider decision-making regarding high-risk infants, including those already having a positive peanut SPT response but not yet clinically reactive, to receive the benefits noted in the LEAP trial, which might reduce the risk of peanut allergy up to 80 %.

Of note, since children with lesser risk factors for peanut allergy were excluded from enrollment in the LEAP trial, there are no prospective, randomized data investigating the benefit or risk of early peanut introduction in the general to low-risk populations. Consequently, this communication’s guidance is limited to applying the findings of the LEAP trial to other similar high-risk children in more diverse settings around the world. However, multiple guidelines have not recommended delaying allergen introduction in the general to low-risk populations.

## Interim guidance regarding early peanut introduction

Based on data generated in the LEAP trial and existing guidelines, the following interim guidance is suggested to assist the clinical decision-making of health care providers:

- There is now scientific evidence (*Level 1* evidence from a randomized controlled trial) that healthcare providers should recommend introducing peanut-containing products into the diets of “high-risk” infants early on in life (between 4 and 11 months of age) in countries where peanut allergy is prevalent because delaying the introduction of peanut can be associated with an increased risk of peanut allergy.
- Infants with early-onset atopic disease, such as severe eczema, or egg allergy in the first 4–6 months of life (see Box 1 for example LEAP criteria), might benefit from evaluation by an allergist or physician trained in management of allergic diseases in this age group to diagnose any food allergy and assist in implementing these suggestions regarding the appropriateness of early peanut introduction. Evaluation of such patients might consist of performing peanut skin testing, in-office observed peanut ingestion, or both, as deemed appropriate after discussion with the family. The clinician can perform an observed peanut challenge for those with evidence of a positive peanut skin test response to determine whether they are clinically reactive before initiating at-home peanut introduction. Both such strategies were used in the LEAP trial protocol.
- Adherence in the LEAP trial was excellent (92 %), with infants randomized to consume peanut ingesting a median of 7.7 g peanut protein (interquartile range: 6.7 – 8.8 g) per week during the first 2 years of the trial compared with a median of 0 g in the avoidance group (see Box 2 for examples of peanut-containing foods used in the LEAP trial). Although the outcome of the LEAP regimen was excellent, the study does not address use of alternative doses of peanut protein, minimal length of treatment necessary to induce the tolerogenic effect, or potential risks of premature discontinuation or sporadic feeding of peanut.

## Rationale for evaluating and applying this policy to a high-risk population

The LEAP trial demonstrates that early peanut introduction can be successfully carried out in a high-risk population, such as the population defined in the LEAP trial. However, without intervention by health care providers, there is the potential that such high-risk infants will remain at risk for delayed introduction of solids and allergenic foods into their diet because of the widespread belief that such foods may exacerbate eczema.

There will be more extensive guidelines in the near future from the NIAID Working Group and EAACI Guidelines Group with their multidisciplinary stakeholders. These groups will consider all the available data and determine whether there is sufficient evidence to apply prevention strategies to the general population. However, engagement of the primary care, allergy, and dermatology communities to rapidly implement these findings and change the culture of early feeding practices is essential, and the forthcoming NIAID Working Group’s and EAACI Guidelines Group’s documents will better clarify a best-practices approach.

## Box 1. Enrollment criteria used in the LEAP trial

**Infants considered at “high risk” as defined by the LEAP trial criteria:**

*Egg allergy*: Children with either –

1) A SPT wheal diameter ≥6 mm from exposure to raw hen’s egg white and no history of previous egg tolerance

OR

2) A SPT wheal diameter ≥3 mm from exposure to pasteurized hen’s egg white and allergic symptoms related to exposure to hen’s egg

*Severe eczema:* An eczematous rash that:

1) Requires application of topical creams, ointments, or both, containing corticosteroids or calcineurin inhibitors, and that, if the participant is <6 months of age, lasted for at least 12 of 30 days on 2 occasions, or, if the participant is >6 months of age, lasted for at least 12 of 30 days on 2 occasions in the last 6 months,

OR

2) Is currently or was previously graded ≥ 40 using the modified SCORAD evaluation

**Example of SPT method used in the LEAP trial**

• SPTs to peanut should be performed in the presence of a negative control and a positive histamine control.

• SPTs should be performed in duplicate, and the maximum wheal diameter of the two SPT responses should be calculated and rounded up to the greatest whole millimeter.

Of note, in the LEAP trial measurement of IgE to peanut resulted in considerably higher rates of sensitization compared with skin testing, which could lead to numerous unnecessary oral peanut challenges.

## Box 2. Examples of peanut-containing foods used in the LEAP trial

• Smooth peanut butter (1 teaspoon) mixed with milk or with mashed or pureed fruit

• *Bamba® snack (Osem; approximately two thirds of a 1 oz. (25 g) bag; 21 sticks of Bamba®)

 - For young infants (<7 months), softened with 20–30 mL water or milk and mixed with milk or with mashed or pureed fruit or vegetables

• Peanut soup

• Finely ground peanuts mixed into other foods such as yogurt

*Other foods more customary to particular nations/cultures may be substituted

*Whole peanut is not recommended for introduction because this is a choking hazard in children less than 4 years of age*

## Abbreviations

LEAP: Learning Early about Peanut Allergy
NIAID: National Institute of Allergy and Infectious Diseases
EAACI: European Academy of Allergy and Clinical Immunology
SPT: Skin prick test
ITT: Intention-to-treat
NNT: Number needed to treat
